# A Dynamical Analysis of the Suitability of Prehistoric Spheroids from the Cave of Hearths as Thrown Projectiles

**DOI:** 10.1038/srep30614

**Published:** 2016-08-10

**Authors:** Andrew D. Wilson, Qin Zhu, Lawrence Barham, Ian Stanistreet, Geoffrey P. Bingham

**Affiliations:** 1 School of Social Sciences, Leeds Beckett University, Leeds, UK; 2 Division of Kinesiology and Health, University of Wyoming, Laramie, WY, USA; 3 School of Archaeology, Classics and Egyptology, University of Liverpool, Liverpool, UK; 4 School of Environmental Sciences, University of Liverpool, Liverpool, UK; 5 Stone Age Institute, Bloomington, IN, USA.; 6 Department of Psychological and Brain Sciences, Indiana University, Bloomington, IN, USA

## Abstract

Spheroids are ball-shaped stone objects found in African archaeological sites dating from 1.8 million years ago (Early Stone Age) to at least 70,000 years ago (Middle Stone Age). Spheroids are either fabricated or naturally shaped stones selected and transported to places of use making them one of the longest-used technologies on record. Most hypotheses about their use suggest they were percussive tools for shaping or grinding other materials. However, their size and spherical shape make them potentially useful as projectile weapons, a property that, uniquely, humans have been specialised to exploit for millions of years. Here we show (using simulations of projectile motions resulting from human throwing) that 81% of a sample of spheroids from the late Acheulean (Bed 3) at the Cave of Hearths, South Africa afford being thrown so as to inflict worthwhile damage to a medium-sized animal over distances up to 25 m. Most of the objects have weights that produce optimal levels of damage from throwing, rather than simply being as heavy as possible (as would suit other functions). Our results show that these objects were eminently suitable for throwing, and demonstrate how empirical research on behavioural tasks can inform and constrain our theories about prehistoric artefacts.

While other animals have been known to throw objects on occasion^1^, none can match the speed, accuracy and distances that a trained human can achieve. Humans are uniquely specialised for throwing, both anatomically^3^ and psychologically^4^. This specialisation speaks to the vital role throwing has played in our evolutionary past, both by enabling us to hunt small to large-sized fauna, and to deter and compete with the carnivore guild to access and scavenge carcasses that would otherwise have been out of our predatory reach. The ability to damage or kill prey at a distance not only expands the range of foods available, but also reduces the risk of close confrontation with dangerous prey.

Before the development of throwing spears, our ancestors were faced with the task of finding and using objects suitable for hunting and defence. Imagine a human, searching for an object to throw so as to cause the most damage possible to a prey animal or a competitor. Their job is to find an object of an optimum size and weight; large and heavy enough to fly far and cause damage, but not too large or heavy as to interfere with producing the high speed throw required for distance and damage. This is a perceptual task; the person needs to perceive throwing-relevant *affordance*^5^ properties of objects and be able to discriminate between objects that vary in those properties.

This article applies research on how modern humans perceive the throwing affordances of objects to inform a mathematical analysis of spheroids found at the Middle Pleistocene site of Cave of Hearths, South Africa, in order to evaluate the potential of these objects as projectiles for throwing. We describe these spheroids, and then outline the task dynamical approach to affordance perception that informs current research into how perception-action systems select and control skilled behaviours.

Spheroids were a widespread and potential early projectile in the archaeological record of Africa and Eurasia^6^. Spheroids are ball-shaped stone objects that vary from perfectly round to elliptical or more angular sub-spheroidal forms (Fig. 1a). They are the product of human action through percussion, or they can be naturally shaped stones that have been selected and transported by humans for use (manuports)^7^,^8^. They are found at African archaeological sites in levels dating from 1.8 million years ago (Early Stone Age) to at least 70,000 years ago (Middle Stone Age) making them one of the longest-used technologies^9^.

The function of spheroids has been much discussed and tested experimentally. Theories about their function include use as bolas stones for hunting^10^, as the by-products of the extended use as hammer stones for making stone tools^11^,^12^,^13^, as grinding stones for processing plant material^6^, as tools for breaking open bones^14^, and tools for re-sharpening grinding slabs^15^. The use of spheroids for preparing grinding surfaces continued into the recent past among farming and hunter-gatherer communities^16^,^17^,^18^. There is a trend in the archaeological record for Middle Stone Age spheroids to be rounder than in the Early Stone Age (ESA) which may reflect a change in use or production methods^13^.

Their spherical shape and weight also make them potentially useful projectiles for hunting and self-defence^14^,^18^. In her review of ethnographic and historic accounts of stone throwing, Isaac^14^ notes the efficacy of rounded stones when thrown overhand as missiles for hunting and as weapons. Roundedness is the key feature in the selection of stones, whether unmodified or deliberately shaped, and in this study we investigate the suitability of rounded stones as thrown missiles.

Our data is drawn from the site of the Cave of Hearths, Makapans Valley, South Africa (24°08′25″S, 29°12′00″E) which is known for its long archaeological record that preserves intervals of occupation from the late Acheulean (ESA) to the Iron Age^18^. Large-scale excavations by Mason in 1953–1954 demonstrated the association of spheroids with the late Acheulean (Beds 1–3) which is estimated to be no older than 500,000 years ago^19^. Bed 3 contains the largest number of Acheulean artefacts among these lower beds, which include handaxes, cleavers, scrapers, denticulates and spheroids^20^,^21^. The artefacts are preserved in breccia which incorporates a mix of fine colluvial sediments and coarser clasts derived from within the cave^19^,^22^. A partial human mandible was also recovered from Bed 3 and has been attributed to *Homo sapiens rhodesiensis*^23^ (now *H. heidelbergensis* by association with the Broken Hill cranium, Kabwe, Zambia^24^), or to *Homo sapiens sapiens*^25^. The late Acheulean at the Cave of Hearths may just pre-date the invention of stone-tipped spears in the region^26^,^27^,^28^. This adds interest to the study of spheroids as possible missiles, especially since they continued to be brought to the site in the Middle Stone Age, though in reduced numbers^21^.

The Bed 3 spheroids (n = 227) are unusual in that they are dominated by diabase (n = 166), an intrusive igneous rock which weathers naturally into spheroidal shapes of varying sizes (Fig. 1b)^21^. Diabase outcrops locally in dykes with the nearest 2 km from the cave^21^,^29^. The absence of a diabase source in the cave system supports Mason’s interpretation that the spheroids were selected for their size and shape and transported to the site^21^. The importance of this diabase sample lies in the observation that the objects have not been modified by use, unlike the minority of spheroids in Bed 3 which are made on other rock types (quartzite, quartz, chert, granite) and which show signs of percussion damage^21^. The absence of damage on the diabase spheroids is the basis of Mason’s speculation that these artefacts were missiles “intended for throwing”^21^.

To assess the potential use of these objects as projectiles we extracted data on spheroid dimensions and weight (Appendix III^6^) from a partial sample of the Cave of Hearths Bed 3 assemblage. Willoughby examined 155 spheroids of which 150 were diabase. She also calculated indices of sphericity (on a scale of 0.01 to 1.00) and smoothness (extent of shaping the surface ranging from 0.1 to 0.9). We restricted our sample to those diabase spheroids towards the more rounded end of the spectrum for each index (≥0.7) to assess Isaac’s observation about the efficacy of this shape as a potential missile and to simplify the simulations. The resulting sub-set totalled 55 spheroids.

In order to evaluate how suitable these objects are for throwing, we need a way of quantifying that suitability, and so we now turn to the topic of affordances, and in particular research on the perception of the affordances of objects for throwing.

An affordance is an action-relevant property of an object in the context of a task. My coffee cup affords being grasped by me, because it has a size and shape that offer a surface I can enclose with my hand. The term was coined by James J Gibson^5^ as part of his ecological approach to perception and action. He proposed that organisms can perceive the affordances of our environments and use that perception to produce functional behaviour that complements the opportunity for action the affordance describes. Since the idea was first proposed, there has been a great deal of research demonstrating that people do, indeed, perceive the affordances of their environments and use these to coordinate and control their behaviour^30^.

The perceptual task facing hominins during the Pleistocene was to select an object from a range of alternatives that best afforded being thrown with great speed. In recent years several of us have explored the perception of the affordances of objects for throwing from a *task dynamical* perspective^31^,^32^,^33^,^34^,^35^,^36^.

Skilled, functional behaviour entails moving so as to complement the local task demands. Tasks are most completely described at the level of *dynamics*^37^. In order to evaluate a behaviour, we therefore need to characterise the task dynamics that the behaviour is complementing. For throwing, the relevant dynamics are those of projectile motion. The goal of throwing is to produce a projectile (ballistic) trajectory that satisfies some task demand (maximising distance, intercepting a target, or inflicting damage to that target). A throw is skilled and functional if it is selected and executed with respect to these dynamical task demands, and an object affords throwing if it is dynamically suited to meeting those demands.

The most studied task demand is choosing projectiles for throwing to maximum distance^31^,^32^,^33^,^34^,^35^,^36^. The dynamical projectile properties that affect distance travelled are the size and the weight. In the context of throwing, for a given size, there is an optimal weight that produces a maximum distance. Thrown distance decreases for objects both lighter and heavier than the optimum, and the specific optimal weight changes as the object size changes. The affordance of ‘throwability to a maximum distance’ is therefore this particular relation between size and weight. The perceptual question is then, can people perceive which size-weight combinations best afford throwing to a maximum distance?

Research^31^,^32^ has investigated this using spherical objects that varied independently in size and weight. Participants hefted the objects in their hands and ranked the objects in the order in which they judged they could throw them for distance (imagine standing on the shore of a lake, picking up and hefting stones until you feel one that seems as if it will really fly). They were then later asked to throw those objects. People’s judgments were both confident and accurate (i.e. they threw their preferred choices the farthest). They can perceive the affordance. Follow-up work has shown that people can also readily judge relative throwability, and so can still pick the best object from a set even if that set doesn’t contain the globally best object^35^. This affordance perception is based on the perception of felt heaviness^36^ (the different size-weight combinations that best afford throwing for distance feel equally heavy), and it is the characteristics of this perceptual information which enables selecting the appropriate objects (and produces the size-weight illusion^4^).

Throwing in a hunting or scavenging context means throwing to inflict damage. Damage is proportional to the impact speed, which in turn depends on the release speed. Release speed is maximised when throwing an object that best affords maximum distance throwing, and the perceptual research demonstrates that these objects can be identified ahead of time by any person experienced in throwing^31^,^32^,^35^. Given a set of objects (say, by a riverbed) then the relatively best suited ones for throwing can be identified and selected. If a set of objects found in an archaeological site have been selected (or shaped) to be used as thrown projectiles in hunting or scavenging, we would expect the size-weight distribution of the set to be strongly biased towards combinations that best afford being thrown for a maximum distance. The current project tests this hypothesis for the set of 55 objects from the Cave of Hearths site described above.

We began with the sizes and weights of the spheroids. We then simulated spherical objects of these sizes and weights being thrown by humans, using a simulation of projectile motion with quadratic drag parameterised by research on humans throwing objects that vary in these properties. These simulations gave us the maximum release speeds and distances produced when these objects are thrown. The simulations also gave us impact speeds at various points in the trajectory, which enabled us to estimate the amount of damage each spheroid would cause if thrown to hit to a small-medium sized prey animal. Finally, we characterised the size-weight distribution of this set of objects relative to how well they afford being thrown. The results show that the majority of these 55 objects from the Cave of Hearths are ideally suited for throwing so as to stand a high probability of inflicting significant damage to an animal over long distances.

## Methods

As described, our raw data was about a set of objects from the Cave of Hearths site. To simplify the simulations, we restricted our sample to the 55 spherical diabase spheroids (minimum sphericity 0.7, mean 0.8, SD 0.08) and their sizes (geometric mean diameter) and weights were the initial parameters for the simulations described below.

The goal of the simulations (implemented in Matlab R2014b; Mathworks, MA) was to evaluate the amount of damage these objects could achieve if thrown. We therefore began with our measure of damage and then worked backwards to generate all the information we required to compute it.

### Damage Index: Blunt Criterion

Our chosen measure of damage was the *Blunt Criterion* (BC)^38^. This is an energy-based measure that can be used to predict the amount of injury from a given blunt projectile. The equation is


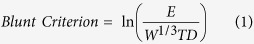


where E is the kinetic energy (1/2MV^2^) of the projectile on impact in joules, M is the projectile mass in kilograms, V is the impact velocity in meters per second, W is the effective mass of the individual in kilograms, T is the thickness of the body wall being hit, in centimetres and D is the effective projectile diameter in centimeters. This ratio is a measure of the amount of energy delivered expressed in terms of the body’s ability to absorb that energy, and it allows us to directly evaluate the likelihood of an impact causing damage.

In order to compute how much damage each spheroid could inflict, we needed to estimate the impact velocity of each spheroid when thrown over some distance, and the parameters for the denominator. We have assumed an expert thrower targeting a prey animal like an impala with an overhand throw for the simulations described below. We chose this animal to represent the kind of small to medium sized antelope (bovid size class 2, 23–84 kg live weight) that is well-represented in the Cave of Hearths faunal assemblage and for which there is evidence that humans were actively involved in creating the assemblage^39^. (Parameters for the impala target taken from http://gadi.agric.za/articles/Furstenburg_D/impala.php).

### Impact Velocity

Computing impact velocity requires simulating the projectile motion that the spheroid undergoes when thrown to a given distance. Projectile motion takes three input parameters; a release height, release velocity and release angle. We needed parameters suitable for throws made by a human contemporary with the Middle Pleistocene age of the Cave of Hearths sample, so we looked to previous research to estimate these numbers.

#### Release height

Carretero *et al*.^40^ reported an average height of 1.63 m for *Homo heidelbergensis* in Europe, while Trinkaus^41^ reported an individual of the same species from closer to the Cave of Hearths (Broken Hill (Kabwe), Zambia) with a height of 1.79 m. The humans likely to have been using these spheroids were therefore about as tall as modern *Homo sapiens*, so for the simulations we set the thrower height to the modern average male human height of 1.75 m, and the release height to 2 m to include the arm length in an overhand throw. The relevant height for the simulations is actually the difference between the release and landing heights. Impala stand approximately 0.85 m at the shoulder, so the difference is 1.15 m.

#### Release velocity

Damage is maximised when release velocity is maximised. Throwers maximise their release velocity when throwing for maximum distance and this velocity has been empirically shown to vary as a power law function (V = kW^n^) of object mass^32^,^34^,^42^. Zhu & Bingham^32^ plotted mass vs. release velocity from expert throwers and fitted a regression line to estimate the power law parameters. For objects with mass >0.05 kg the power law was Velocity = 14.8 * Weight^−0.15^. This matched Cross^42^, who found the exponent was equal to −0.15 for objects of mass <0.73 kg and −0.4 for heavier objects. This gave us an estimate of the maximum release velocity a human could produce for each object; we set k to 14.8 and n to either −0.15 or −0.4, as required by the spheroid mass.

#### Release angle

For a given release height and release velocity, the distance travelled varies as a function of the release angle. We therefore ran simulations of projectile motion with quadratic drag using our release height and computed velocity. We computed the distance travelled for each release angles varying from −90° to 90° in 1° increments and identified a) the maximum distance a person could throw each object and b) the angle that produced that distance. We then computed three proportions of that maximum distance (1/3, 1/2, and 2/3) and identified the release angle from the simulations that produced that distance, given the release height and release velocity from above.

We now had a full set of release parameters for each spheroid for our simulations; a release height of 1.15 m, release velocities that varied as a power law function of the object mass and maximised distance, and release angles that took those throws to various proportions of the maximum distance. We then used these parameters to simulate the projectile motion the objects would undergo, which allowed us to calculate the impact velocity at each distance and therefore the energy the spheroid would impart to the target.

### BC Denominator Values

#### Effective mass (W)

This is the mass of the rigid body segment being struck. For these simulations we have assumed that the target was a prey animal, something like an impala (average mass 57 kg). The likely target for a throw would be the animal’s torso (the largest target area). The head + trunk segment of a wide range of animals constitutes ~70% of the total body mass^43^, so W was set to 57*0.7 = 39.9 kg.

#### Body wall thickness (T)

This is the thickness of the torso covering the internal organs (primarily the ribs) and it can be estimated as T = kW^1/3^. We chose the empirically measured value of k for steer (k_thorax_ = 0.634), resulting in T = 8.43^38^.

#### Effective diameter of impact (D)

The effective diameter of impact depends upon the radius of the object relative to the body wall thickness. If the diameter is greater than 2T then the area of contact A is A = πT(D − T); D > 2T, and the effective diameter used in the damage calculation is 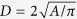. For objects where D < 2T, the effective diameter used was the actual diameter^38^.

For all objects, we were now able to compute the Blunt Criterion value for an impact at four distances. The final task was to quantify, in practical terms, how much damage an impact resulting in a given BC would inflict.

### Probability of causing increasing severity of injury

Sturdivan *et al*.^38^ regressed the BC for various objects against the severity of the injury inflicted from a variety of data sets. Injury severity was assessed using the Abbreviated Injury Scale (AIS)^44^. This is a 6 point scale for classifying and describing the severity of injuries, where AIS 1 is a minor laceration with a 0% chance of death, and AIS 6 is a fatal injury (100% chance of death).

Table 3 of Sturdivan *et al*.^38^ reports the regression parameters *a* and *b* obtained for each AIS level, and they used these to create an equation we can use to estimate the probability of an impact with a given BC producing AIS levels 2–6 as


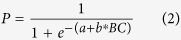


For each spheroid, we took the computed BC and the relevant parameters *a* and *b* and calculated the probability of that object producing an injury at each AIS level. We then computed the proportion of spheroids in the data set that had a >50% chance of inflicting AIS injury levels 2–6.

## Results

**Figure 2:**
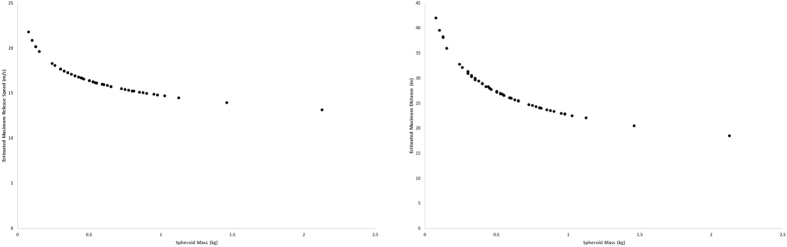
Scatterplots of how maximum release speed (A) and maximum distance (B) varied with mass in the simulations.

For each spheroid we simulated the projectile motion given an estimated release height and an estimated maximum release speed for release angles ranging from −90° to 90°. We then identified which release angle produced the maximum distance; the modal angle was 43°. Simulated release speeds and the resulting maximum distances are plotted against spheroid mass in Fig. 2a,b respectively. These objects could readily be projected at high speeds (typically 15–20 ms^−1^) and over useful distances (typically between 25–35 m).

An impact occurring at maximum distance is the worst case scenario; the impact velocity will be lowest and the resulting damage minimal. We therefore characterised the probabilities of each spheroid causing various levels of injury at this distance to establish the lower bound on their potential as projectile weapons.

**Figure 3:**
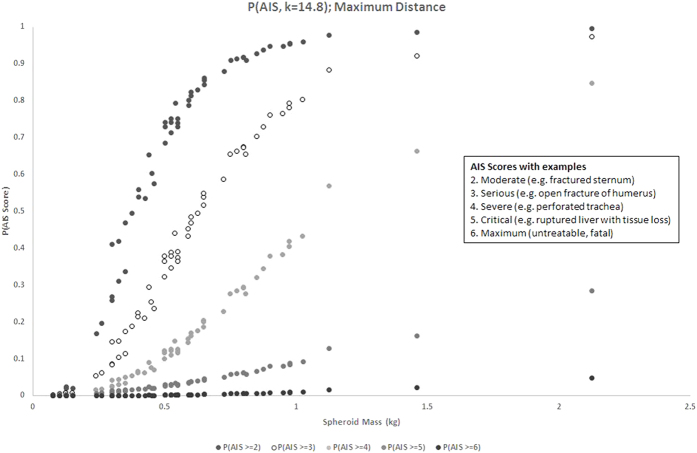
The probability that spheroids of various masses would inflict an injury of AIS score 2–6 for an impact at the maximum distance the simulations suggested they could be thrown (the worst case scenario).

We computed the Blunt Criterion for each spheroid on impact at maximum distance on the torso of an impala sized animal. We used the regression coefficients^38^ to estimate the probability of that impact causing varying levels of damage. Figure 3 plots the probability of each object causing damage rated AIS 2 or higher, 3 or higher, 4 or higher, 5 or higher, and 6.

Most of the spheroids have a good probability of causing useful damage (AIS = 2 or more; grey dots) for an impact occurring at maximum distance. As an indication, 75% of the spheroids had a greater than 50% chance of incurring such damage, while 42% of the spheroids had an equivalent probability of incurring an AIS > =3 injury. This number falls to 5% for AIS > =4 and 0% above that. None of the spheroids could kill an impala, but most of them would inflict significant damage if they hit, even at maximum distance. The disabled animal could then be dispatched with a suitable weapon (eg, club, spear, stone) or run to death through blood loss and exhaustion^45^, and of course multiple spheroids could be thrown at the animal as well.

**Figure 4:**
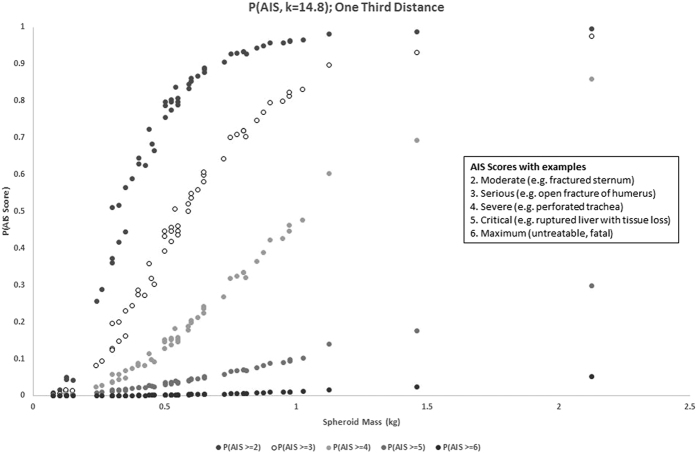
The probability that spheroids of various masses would inflict an injury of AIS score 2–6 for an impact at one-third of the maximum distance the simulations suggested they could be thrown (the best case scenario that we simulated).

**Figure 5:**
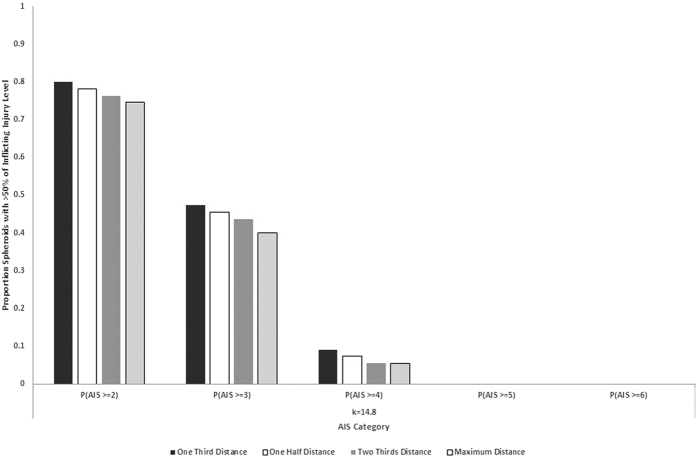
The proportion of spheroids that could cause AIS score 2–6 level injuries. Bars represent the different simulated distances.

We repeated the analysis for impacts at closer distances; the basic result was replicated but the odds of damage increased as distance came down (impact velocities are higher for throws to less than the maximum achievable distance). Figure 4 shows the probability of inflicting various levels of damage for throws to one-third of maximum distance (at least 6 m but typically between 8–10 m). This was the best case scenario that we simulated. Figure 5 summarises the proportion of spheroids that had a greater than 50% chance of incurring various levels of damage for various proportions of maximum distance (1/3, 1/2, 2/3 and 1).

**Figure 6:**
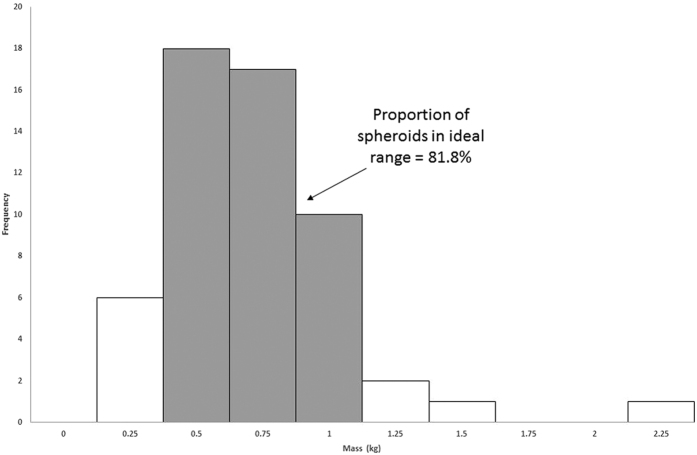
The distribution of the 55 spheroid masses in our sample. The majority of objects (grey bars) fall in a region where thrown objects cause ideal damage without being too heavy for throwing far.

Damage increases with mass, but heavier spheroids are bigger (given the constant density) and therefore harder to throw. There is therefore a trade-off between damage potential and mass when filtered through the dynamics of throwing. Figures 3 and 4 reveal a region (approximately 0.75 kg +/− 0.25 kg) where the amount of damage inflicted approaches the highest it will get. Figure 6 shows a histogram of the distribution of masses in the Cave of Hearths sample. 81.8% of the sample masses fell into this range.

In summary, the majority of these objects are of an ideal size and weight to be thrown so as to cause functionally significant damage to a small-medium prey animal over long distances.

## Discussion

Using research on the perception of affordances for maximum distance (and therefore maximum speed and damage) throwing, we simulated the projectile motions a sample of 55 spheroids would undergo if thrown by an expert. We then used these simulations to estimate the probability of these projectiles causing damage to a medium size prey animal, and found that a majority of the objects afford causing useful amounts of damage over quite large distances. These observations lend biomechanical support to the argument that the throwing of stones, and in particular spheroids, played a key role in the evolution of hunting before the development of spears^13^. We are not claiming this to be the sole or even primary function of spheroids, but these results show that this function is an option that warrants reconsidering as a potential use for this long-lived, multi-purpose tool.

The majority (81%) of the objects have masses in a range that produce nearly maximum damage while remaining throwable. This range does include the less than optimal mass of 0.5 kg. This probably reflects the fact that these objects were being found, not crafted, and there is no guarantee of finding an actually optimal object in a given location. Humans can accurately perceive *relative* throwability, however^35^ and the structure of the sample reflects the best spheroids for throwing they could find locally.

The analysis presented here is a proof-of-concept for applying the tools and concepts of the task dynamical approach to affordances^30^ to studying the functions of prehistoric artefacts. We limited our analysis in a variety of ways in order to simplify and enable the simulations, but these limits are not limits on the approach itself. For example, we restricted ourselves to simulating spherical objects because the relevant throwing research involved spherical objects and we depended on that data for the parameters via the empirically established power law relationship between projectile mass and release velocity. The needs of this analysis can therefore drive future empirical research using less spherical objects, such as those found in older Stone Age assemblages in East Africa, to establish the various parameters. We also made some decisions about the nature of the target (here, an impala sized animal); modelling other targets (e.g. other animals) is simply a matter of changing these parameters. We chose a medium sized animal as a plausible large target given their prevalence in this particular archaeological site (the larger the animal, the better its ability to absorb the force of impact and the lower the resulting BC) and the spheroids performed very well.

We propose that this task dynamical affordance framework has much to offer the study of prehistoric artefacts, especially when the function of those artefacts remains ambiguous. Spheroids, for example, potentially suit a wide variety of purposes; this analysis shows that they are definitely well suited for one of these (throwing). Quantifying the dynamics of the alternative functions (e.g. as hammer stones) then allows formal descriptions of the affordances of the tasks, which can then drive the necessary empirical and modelling work to identify whether a given object also offers that affordance and to what extent. It can also drive the critical work of establishing whether people can perceive that affordance, and if so, how^36^. Behaviour is shaped by the form of our perceptual contact with the world, and understanding both the dynamical objects of perception (affordances) and the information for those affordances is critical to understanding why a given behaviour (e.g. selecting one spherical rock rather than another one) happens the way that it does^46^.

## Additional Information

**How to cite this article**: Wilson, A. D. *et al*. A Dynamical Analysis of the Suitability of Prehistoric Spheroids from the Cave of Hearths as Thrown Projectiles. *Sci. Rep.* **6**, 30614; doi: 10.1038/srep30614 (2016).

## Figures and Tables

**Figure 1:**
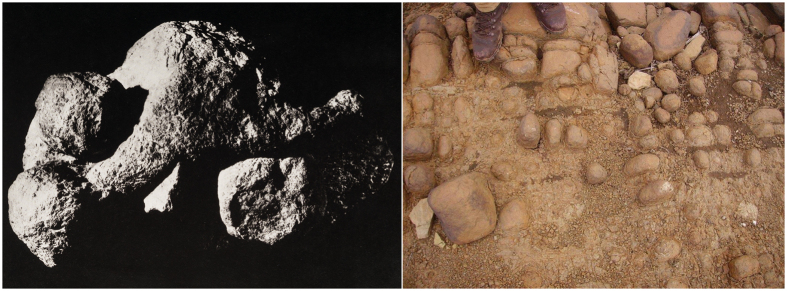
(**a**) (left) Diabase spheroids in breccia from Bed 3, Cave of Hearths. (Image originally published in Mason 1988 (Plate 40) and re-used with the permission of Revil Mason who retains copyright). (**b**) (right) Diabase outcrop near the Cave of Hearths, South Africa, showing a rectangular jointing pattern within which there is spheroidal weathering. (Note the distribution of sizes and shapes, with some more spherical than others confirming the need to select suitable projectiles from a varied set). Photo courtesy and copyright of Judy Maguire.

## References

[b1] Goodall J. The Chimpanzees of Gombe: Patterns of Behavior. Cambridge: Harvard University Press (1986).

[b2] Westergaard G. C. & Suomi S. J. Aimed throwing of stones by tufted capuchin monkeys (Cebus apella). Hum Evo. 9(4), 323–329 (1994).

[b3] Roach N., Venkadesan M., Rainbow M. & Lieberman D. Elastic energy storage in the shoulder and the evolution of high-speed throwing in Homo. Nature 498, 483–486 (2013). 23803849 10.1038/nature12267 PMC3785139

[b4] Zhu Q. & Bingham G. Human readiness to throw: the size/weight illusion is not an illusion when picking the best objects to throw. Evol. Hum. Behav. 32, 288–293 (2011).

[b5] Gibson J. J. The ecological approach to visual perception. Boston: Houghton Mifflin. (1979).

[b6] Willoughby P. R. Spheroids and battered stones in the African Early and Middle Stone Age. Oxford: BAR International Series 321 (1987).

[b7] Clark J. D. The stone ball: its association and use by prehistoric man in Africa. In Balout L. (ed.), *Congrès Panafricain de Préhistoire, II, Alger, 1952*. Paris: Arts et Métiers Graphiques, pp. 403–417 (1955).

[b8] Leakey M. D. Olduvai Gorge: Excavations in Beds I and II, 1960–1963. Cambridge: Cambridge University Press (1971).

[b9] Barham L. & Mitchell P. The First Africans: African Archaeology from Earliest Toolmakers to Most Recent Foragers. Cambridge: Cambridge University Press (2008).

[b10] Leakey L. The bolas in Africa. Man 48, 48 (1948).

[b11] Bordaz J. *Tools of the old and new stone age*. (Published for the American Museum of Natural History [by] the Natural History Press. (1970).

[b12] Jones P. R. Results of experimental work in relation to the stone industries of Olduvai Gorge. In Olduvai Gorge, Vol. 5: *Excavations in Beds III, IV and the Masek Beds*, 1968–1971 (Leakey M. ed.) 254–298 (Cambridge University Press) (1995).

[b13] Schick K. & Toth N. *The cutting edge: new approaches to the archaeology of human origins*. Gosport, IN: Stone Age Institute Press (2009).

[b14] Isaac B. Throwing and human evolution. Afr Archaeol Rev 5, 3–17 (1987).

[b15] Van Peer P., Rots V. & Vroomans J. A story of colourful diggers and grinders. Before Farming 2004, 1–28 (2004).

[b16] Reynolds B. The Material Culture of the Peoples of the Gwembe Valley. Manchester: Manchester University Press (1968).

[b17] McCarthy F. D. Australian Aboriginal stone implements. Sydney: Australian Museum Trust (1976).

[b18] Clark J. D. The Prehistory of Africa. London: Thames & Hudson (1970).

[b19] Herries A. & Latham A. Archaeomagnetic studies at the Cave of Hearths. In McNabb J. & Sinclair A. (eds) The Cave of Hearths: Makapan Middle Pleistocene Research Project: Field Research by Anthony Sinclair and Patrick Quinney, 1996–2001. Oxford: Archaeopress, pp. 59–64 (2009).

[b20] McNabb J. The ESA stone tool assemblage from the Cave of Hearths. In McNabb J. & Sinclair A. (eds) The Cave of Hearths: Makapan Middle Pleistocene Research Project: Field Research by Anthony Sinclair and Patrick Quinney, 1996–2001. Oxford: Archaeopress, pp. 75–104 (2009).

[b21] Mason R. J. *Cave of Hearths, Makapansgat, Transvaal*. Johannesburg: Archaeological Research Unit Occasional Paper No. 21, University of the Witwatersrand (1988).

[b22] Latham A. & Herries A. The formation and sedimentary infilling of the Cave of Hearths and Gwaša/Historic Cave Complex, Makapan, South Africa. In McNabb J. & Sinclair A. (eds) *The Cave of Hearths: Makapan Middle Pleistocene Research Project: Field Research by Anthony Sinclair and Patrick Quinney*, 1996–2001. Oxford: Archaeopress, pp. 49–58 (2009).

[b23] Tobias P. J. Human skeletal remains from the Cave of Hearths, Makapansgat, Northern Transvaal. American Journal of Physical Anthropology 34, 335–367 (1971). 5120551 10.1002/ajpa.1330340305

[b24] Buck L. T. & Stringer C. B. Homo heidelbergensis. Current Biology 24 **(6)**, R214–R215 (2014). 24650901 10.1016/j.cub.2013.12.048

[b25] Curnoe D. The mandible from Bed 3, Cave of Hearths. In McNabb J. & Sinclair A. (eds). The Cave of Hearths: Makapan Middle Pleistocene Research Project: Field Research by Anthony Sinclair and Patrick Quinney, 1996–2001. Oxford: Archaeopress, pp. 138–149 (2009).

[b26] Wilkins J., Schoville B. J., Brown K. S. & Chazan M. Evidence for early hafted hunting technology. Science 388, 942–946 (2012). 23161998 10.1126/science.1227608

[b27] Wilkins J., Schovill B. J., Brown K. S. & Chazan M. Kathu Pan 1 points and the assemblage-scale, probabilistic approach: A response to Rots and Plisson, “Projectiles and the abuse of the use-wear method in a search for impact” Journal of Archaeological Science 54, 294–299 (2015).

[b28] Rots V. & Plisson H. Projectiles and the abuse of the use-wear method in a search for impact. Journal of Archaeological Science 48, 154–163 (2014).

[b29] Maguire J. An overview of the physical setting of Makapan. In McNabb J. & Sinclair A. (eds) The Cave of Hearths: Makapan Middle Pleistocene Research Project: Field Research by Anthony Sinclair and Patrick Quinney, 1996–2001. Oxford: Archaeopress, pp. 29–48 (2009).

[b30] Wilson A. D., Weightman A., Bingham G. P. & Zhu Q. Using task dynamics to quantify the affordances of throwing for long distance and accuracy. Journal of Experimental Psychology: Human Perception and Performance. (in press). 10.1037/xhp0000199 26766510

[b31] Bingham G. P., Schmidt R. C. & Rosenblum L. D. Hefting for a maximum distance throw: A smart perceptual mechanism. J Exp Psychol: Hum Percep Perf. 15 **(3)**, 507–528 (1989). 10.1037//0096-1523.15.3.507 2527959

[b32] Zhu Q. & Bingham G. Is hefting to perceive the affordance for throwing a smart perceptual mechanism? J Exp Psychol: Hum Percep Perf. 34, 929–943 (2008). 10.1037/0096-1523.34.4.929 18665736

[b33] Zhu Q. & Bingham G. P. Learning To Perceive the Affordance for Long-Distance Throwing: Smart Mechanism or Function Learning. J Exp Psychol: Hum Percep Perf. 36 **(4)**, 862–875 (2010). 10.1037/a0018738 20695705

[b34] Zhu Q., Dapena J. & Bingham G. P. Learning to throw to maximum distances: Do changes in release angle and speed reflect affordances for throwing? Hum Move Sci. 28 **(6)**, 708–725 (2009). 10.1016/j.humov.2009.07.005 19703718

[b35] Zhu Q., Mirich T. & Bingham G. P. Perception of relative throw-ability. Exp Brain Res. 232 **(2)**, 395–402 (2014). 24162865 10.1007/s00221-013-3747-2

[b36] Zhu Q., Shockley K., Riley M. & Bingham G. P. Felt heaviness is used to perceive the affordance for throwing, but rotational inertia does not affect either. Exp Brain Res. 224, 221–231 (2013). 23099549 10.1007/s00221-012-3301-7

[b37] Bingham G. P. Dynamics and the problem of visual event recognition. In Port R. & van Gelder T. (eds), Mind as Motion: Dynamics, Behavior and Cognition, (pp. 403–448). Cambridge, MA: MIT Press (1995).

[b38] Sturdivan L. M., Viano D. C. & Champion H. R. Analysis of injury criteria to assess chest and abdominal injury risks in blunt and ballistic impacts. J Trauma Acute Care Surg. 56 **(3)**, 651–663 (2004). 10.1097/01.ta.0000074108.36517.d4 15128140

[b39] Ogola C. The taphonomy of the Cave of Hearths Acheulean faunal assemblage. In McNabb J. & Sinclair A. (eds) The Cave of Hearths: Makapan Middle Pleistocene Research Project: Field Research by Anthony Sinclair and Patrick Quinney, 1996–2001. Oxford: Archaeopress, pp. 65–74 (2009).

[b40] Carretero J. M., Rodríguez L., García-González R., Arsuaga J. L., Gómez-Olivencia A., Lorenzo C. & Quam R. Stature estimation from complete long bones in the Middle Pleistocene humans from the Sima de los Huesos, Sierra de Atapuerca (Spain). Journal of Human Evolution 62(2), 242–255 (2012). 22196156 10.1016/j.jhevol.2011.11.004

[b41] Trinkaus E. The human tibia from Broken Hill, Kabwe, Zambia. PaleoAnthropology 145–165 (2009).

[b42] Cross R. Physics of overarm throwing. American Journal of Physics 72 **(3)**, 305–312 (2004).

[b43] Prothero J. Scaling of bodily proportions in adult terrestrial mammals. American Journal of Physiology-Regulatory, Integrative and Comparative Physiology 262(3), R492–R503 (1992). 10.1152/ajpregu.1992.262.3.R492 1558220

[b44] Association for the Advancement of Automotive Medicine. Abbreviated injury scale; 1990 revision: update **98**, AAAM (1998).

[b45] Liebenberg L. Persistence hunting by modern hunter-gatherers. Current Anthropology 47 **(6)**, 1017–1025 (2006).

[b46] Wilson A. D. & Golonka S. Embodied cognition is not what you think it is. Front in Psychol 4, 58 (2013). 10.3389/fpsyg.2013.00058 PMC3569617 23408669

